# SUMO regulates the activity of Smoothened and Costal-2 in *Drosophila* Hedgehog signaling

**DOI:** 10.1038/srep42749

**Published:** 2017-02-14

**Authors:** Jie Zhang, Yajuan Liu, Kai Jiang, Jianhang Jia

**Affiliations:** 1 Markey Cancer Center, Department of Molecular and Cellular Biochemistry, The University of Kentucky College of Medicine, Lexington, KY 40536, USA

## Abstract

In Hedgehog (Hh) signaling, the GPCR-family protein Smoothened (Smo) acts as a signal transducer that is regulated by phosphorylation and ubiquitination, which ultimately change the cell surface accumulation of Smo. However, it is not clear whether Smo is regulated by other post-translational modifications, such as sumoylation. Here, we demonstrate that knockdown of the small ubiquitin-related modifier (SUMO) pathway components Ubc9 (a SUMO-conjugating enzyme E2), PIAS (a SUMO-protein ligase E3), and Smt3 (the SUMO isoform in *Drosophila*) by RNAi prevents Smo accumulation and alters Smo activity in the wing. We further show that Hh-induced-sumoylation stabilizes Smo, whereas desumoylation by Ulp1 destabilizes Smo in a phosphorylation independent manner. Mechanistically, we discover that excessive Krz, the *Drosophila* β-arrestin 2, inhibits Smo sumoylation and prevents Smo accumulation through Krz regulatory domain. Krz likely facilitates the interaction between Smo and Ulp1 because knockdown of Krz by RNAi attenuates Smo-Ulp1 interaction. Finally, we provide evidence that Cos2 is also sumoylated, which counteracts its inhibitory role on Smo accumulation in the wing. Taken together, we have uncovered a novel mechanism for Smo activation by sumoylation that is regulated by Hh and Smo interacting proteins.

It has long been studied that the Hedgehog (Hh) morphogen controls development processes such as proliferation, embryonic patterning, and cell growth^1^,^2^. It has also been shown that malfunction of Hh signaling, e.g. mutations in the Hh pathway components, causes many human disorders, including several types of cancers^3^,^4^,^5^. One good example is that abnormal activation of Smoothened (Smo), an atypical G protein-coupled receptor (GPCR), results in basal cell carcinoma (BCC) and medulloblastoma^1^,^2^, therefore Smo has been an attractive therapeutic target, exemplified by the newly FDA approved drugs^6^.

Most of what is known about the Hh signaling cascade comes from studies of *Drosophila*, where the pathway was originally identified^7^. Hh receiving system consists of Patched (Ptc) and Smo at the plasma membrane. Smo acts as a signal transducer whose activity is inhibited by Ptc in the absence of Hh. How Ptc inhibits Smo is not completely understood, although recent studies indicate that phospholipids act in between Ptc and Smo in *Drosophila* Hh signaling^8^,^9^,^10^. Binding of Hh to Ptc alleviates Ptc-mediated inhibition of Smo, allowing Smo to activate Cubitus interuptus (Ci)/Gli transcription factors and ultimately induce the expression of Hh target genes, such as *decapentaplegic (dpp*), *patched (ptc)*, and *engrailed (en*)^11^,^12^. The regulation of Smo is obviously a key event in Hh signal transduction.

Among the types of protein-based modifications, phosphorylation and ubiquitination of Smo have been extensively studied. In the absence of Hh, cytosolic Smo is highly unstable because of rapid degradation through both the proteasome- and lysosome-mediated pathways, which involve ubiquitination^13^,^14^,^15^. In a dose-dependent manner, thresholds of Hh promote Smo differential phosphorylation by multiple kinases including PKA, CK1 isoforms, aPKC, CK2, and G protein-coupled receptor kinase 2 (Gprk2)^16^,^17^,^18^,^19^, which induces the dimerization and cell surface accumulation of Smo^1^,^12^,^20^. The stimulation of Hh promotes Smo deubiquitination by ubiquitin-specific protease 8 (USP8), which blocks Smo endocytosis and enhances Smo cell surface accumulation^14^,^15^. Although Smo behaves differently from a typical GPCR, it has been shown that Krz, the *Drosophila* non-visual arrestin^21^, downregulates Smo signaling by promoting Smo internalization and degradation in ubiquitin- and Gprk2-independent manners^15^,^22^. It is possible that Krz downregulates Smo activation through a mechanism in parallel with phosphorylation and ubiquitination.

The small ubiquitin-like modifier (SUMO) is post-translationally conjugated to lysine residues of nuclear proteins as well as cytosolic and plasma membrane proteins, resulting in changes in their transcriptional activity or intracellular trafficking^23^,^24^. Sumoylation is promoted by the SUMO-activating enzyme E1, SUMO-conjugating enzyme E2, and SUMO ligase E3, and the SUMO ligase E3 is responsible to recognize the substrate^25^. As a reversible process, SUMO-protein cleavage, or desumoylation, is carried out by SUMO protease, which is also highly regulated in cellular mechanisms such as nuclear transcription factor regulation and intracellular protein trafficking^26^. Interestingly, sumoylation was recently reported to regulate proteins involved in G-protein signaling^27^, however, it is unknown whether the activity of GPCR itself is regulated by SUMO.

To explore the possibility that SUMO regulates Hh signaling proteins, we performed a small scale genetic screen with RNA interference (RNAi) lines. This screen allowed us to determine whether inactivation of the SUMO pathway regulated Hh signaling activity *in vivo*. Further, we were able to examine sumoylation levels of individual Hh signaling component in S2 cells using immunoprecipitation assays. We found that Smo, Costal-2 (Cos2), Fused (Fu), and Ci are all SUMO modified. In this report, we focused on the sumoylation of Smo and Cos2. We further found that the SUMO pathway proteins, including Ubc9 E2 enzyme, PIAS E3 ligase, and Smt3 SUMO isoform, regulate the activity of Smo by elevating the accumulation of Smo in the wing disc and increasing the stability of Smo in S2 cells. In contrast, the ubiquitin-like protease 1 (Ulp1) destabilizes Smo by preventing Smo sumoylation. We further found that Hh promotes Smo sumoylation which activates Smo in a phosphorylation independent manner. Interestingly, we found that Krz inhibits Smo accumulation through blocking Smo sumoylation and that the C-terminal regulatory domain is responsible for Krz inhibitory activity. Finally, we provide evidence that Cos2 is sumoylated, which likely counteracts with its inhibitory activity on Smo accumulation and activation.

## Results

### Sumoylation regulates Hh signaling and Smo activity in *Drosophila* wing

**Figure 1:**
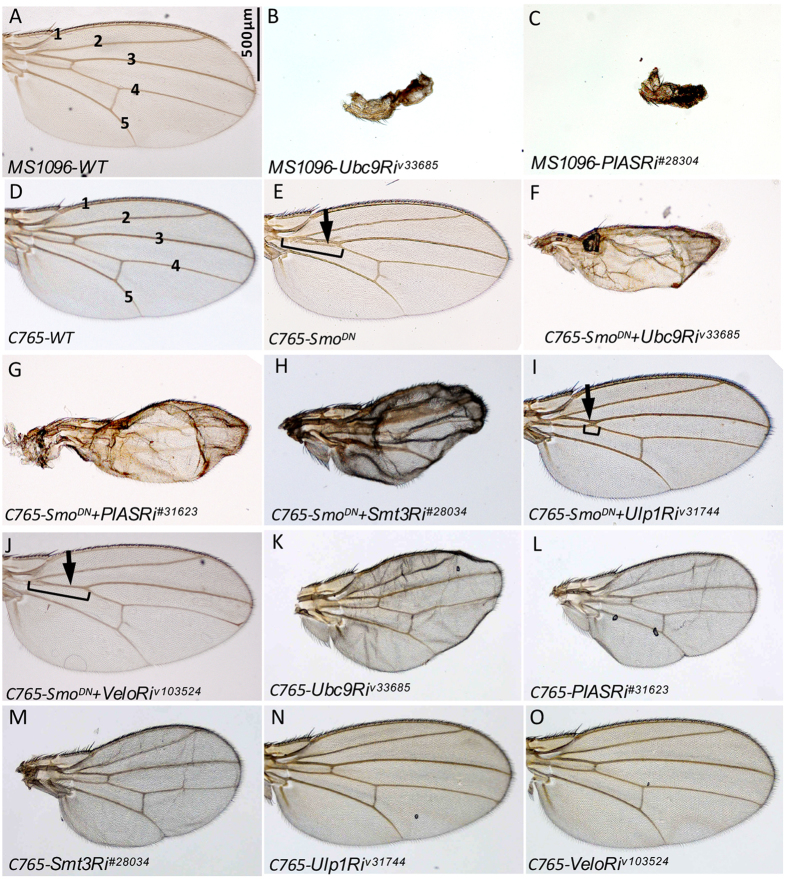
Hh signaling and Smo activity are regulated by sumoylation. (**A**) A wild-type (WT) adult wing from flies with genotype *MS1096*-Gal4-*yw* shows interveins 1–5. Scale bar indicates 500 μm for all adult wing figures. (**B,C**) Abnormal wings shown for the phenotypes caused by Ubc9 and PIAS RNAi using *MS1096*-Gal4. (**D**) A control wing with the genotype of *C765*-Gal4-*yw* shows normal structure of interveins 1–5. (**E**) A wings from flies expressing Smo^−PKA12^ (Smo^DN^) by *C765*-Gal4. Arrow indicates the fusion between Vein 3 and Vein 4 that is a partial loss of Hh phenotype. (**F–I**) Wings from flies expressing Smo^DN^ together with Ubc9 RNAi, PIAS RNAi, Smt3 RNAi, Ulp1 RNAi, or Velo RNAi by *C765*-Gal4. Arrow in I indicates that the fusion phenotype caused by Smo^DN^ is decreased by RNAi of Ulp1. Brackets in E, I, and J indicate the degree of fusion between Vein 3 and Vein 4. (**K–O**) Wing phenotypes from flies expressing Ubc9 RNAi, PIAS RNAi, Smt3 RNAi, Ulp1 RNAi, or Velo RNAi by *C765*-Gal4. Of note, the *C765*-Gal4 is weaker than the *MS1096*-Gal4 therefore mild phenotypes were observed when using C765-Gal4.

To explore whether SUMO pathway plays a role in Hh signaling, we collected RNAi lines from either Vienna Drosophila Research Center (VDRC) or Bloomington Stock Center (BSC) to target SUMO pathway protein expression. We found that inactivation of Ubc9 E2 enzyme or PIAS E3 ligase by RNAi driven by the wing-specific *MS1096*-Gal4 caused severe structural loss in adult wing (Fig. 1B,C, compared to control in Fig. 1A). To examine whether inactivation of the SUMO pathway regulates the activity of Smo, we used a sensitized genetic background by expressing the partial dominant negative Smo^DN^ that we previously described as Smo^−PKA12^ in which two PKA phosphorylation sites were mutated to avoid phosphorylation^28^. Expressing Smo^DN^ by the wing-specific *C765-*Gal4, a weaker Gal4 line than *MS1096*-Gal4, resulted in a partial fusion of Vein 3 and Vein 4, a reproducible phenotype indicative of partial loss of Hh signaling activity (Fig. 1E, compared to WT wing in Fig. 1D). Knockdown of either Ubc9 or PIAS by RNAi in *C765-Smo*^*DN*^ background resulted in small wings with the loss of intervein structures (Fig. 1F,G, compared to Fig. 1E), suggesting that inactivating Ubc9 and PIAS regulates Smo activity and dominantly modifies Smo^DN^ phenotype. Multiple RNAi lines for each gene were tested to make sure the phenotypes were consistent.

SUMO1, SUMO2, and SUMO3 are three isoforms expressed in vertebrates, where SUMO2 and SUMO3 share 97% identity with each other, SUMO1 shares 43% identity with SUMO2 and 3. In *Drosophila*, Smt3 is the single form of SUMO that shares 52% and 73% identity with vertebrate SUMO1 and SUMO2, respectively^29^. We found that RNAi of Smt3 also modified the Smo^DN^ phenotype and resulted in smaller wings (Fig. 1H), suggesting that SUMO may possibly regulate Smo activity in the wing. Protein sumoylation is a dynamic process that often involves a desumoylase. In *Drosophila*, Ulp1 is one of the desumoylases with SUMO-specific protease activity. We found that RNAi of Ulp1 attenuated the activity of Smo^DN^, resulting in a partial rescue of Vein 3 and Vein 4 fusion phenotype (Fig. 1I), further indicating that changes in the expression of SUMO pathway proteins regulate Smo activity in the wing. In contrast, RNAi of Verloren (Velo), the other SUMO protease in *Drosophila*, did not modify Smo^DN^ phenotype (Fig. 1J), indicating the specificity of Ulp1 in regulating Smo. In comparison, RNAi of Ubc9, PIAS, or Smt3 alone by *C765*-Gal4 produced mild phenotype in the adult wing (Fig. 1K–M), and RNAi of Ulp1 or Velo had no effect (Fig. 1N,O), suggesting that the phenotypes shown in Fig. 1F–I were due to the modification of Smo^DN^ activity by inactivation of the SUMO pathway.

### Inactivation of Sumoylation inhibits Smo accumulation by decreasing Smo stability

**Figure 2:**
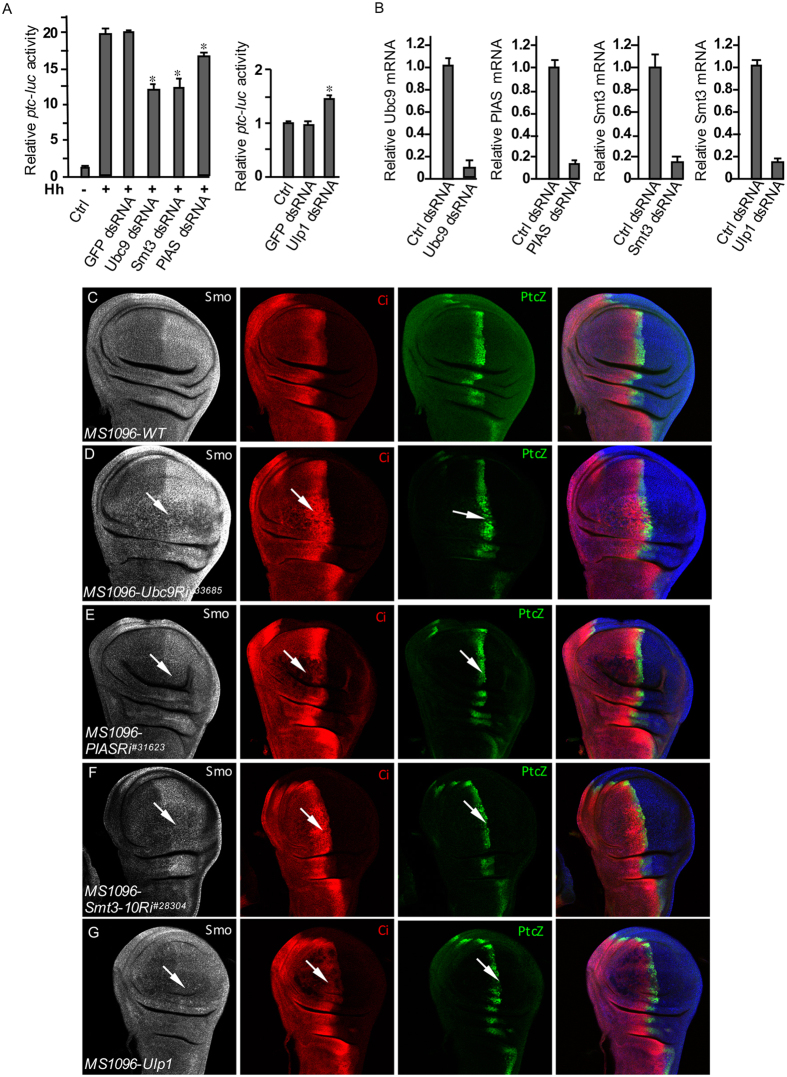
Sumoylation promotes the accumulation and activity of Smo. (**A**) *ptc-luc* reporter assays in S2 cells to examine the Hh signaling activity regulated by the SUMO pathway. Left panel, S2 cells were transfected with tub-Ci and treated with HhN-conditioned medium or control medium, in combination with the indicated dsRNA to knockdown gene expression. The y-axis represents normalized *ptc-luc* activity. *p < 0.001 versus high level of Hh in the second column (Student’s t test). Right panel, S2 cells were transfected with tub-Ci and treated either GFP dsRNA or Ulp1 dsRNA. *p < 0.001 versus GFP dsRNA in the second column (Student’s t test). (**B**) The efficiency of RNAi targeting the indicated genes. (**C**) A WT wing disc from third instar larva was immunostained for Smo, Ci, and *ptc*-*lacZ*. (**D–F**) Wing discs from third instar larvae expressing Ubc9 RNAi, PIAS RNAi, or Smt3 RNAi by the wing-specific *MS1096*-Gal4 were stained for Smo, Ci, and *ptc*-*lacZ*. Arrows in the grey panel indicate Smo accumulation inhibited by RNAi of the E2 and E3. Arrows in the red and green panels indicate the levels of Ci and *ptc*-*lacZ* expression. (**G**) A wing disc over-expressing Ulp1 by *MS1096*-Gal4 was stained for Smo, Ci, and *ptc*-*lacZ*. Arrow in the grey panel indicates the decreased accumulation of Smo. Arrows in the red and green panels indicate the expression of Ci and *ptc*-*lacZ*. All imaginal discs shown in this study were oriented with anterior on the left and ventral on the top. The representative images shown in this study were from five or more images for each experiment.

To further examine the roles of sumoylation in regulating Hh signaling, we carried out a *ptc*-*luciferase (ptc-luc*) reporter assay in S2 cells to monitor Hh pathway activity when using RNAi to inactivate the SUMO pathway. We found that double-stranded RNA (dsRNA) targeting Ubc9, Smt3, or PIAS significantly reduced *ptc-luc* activity induced by the treatment with Hh in cultured S2 cells (Fig. 2A). RNAi of GFP did not change *ptc-luc* activity thus served as a control. dsRNA treatment consistently had high efficiency to knock down gene expression (Fig. 2B).

We wondered whether the inactivation of SUMO pathway could regulate endogenous Smo in the wing, given the fact that RNAi of sumoylation protein expression changed Smo^DN^ activity (Fig. 1). We found that knockdown of Ubc9 by RNAi severely reduced Smo accumulation and attenuated *dpp*-*lacZ* expression in the wing imaginal disc, an early stage of wing development (Fig. 2D, compared to WT immunostaining shown in Fig. 2C). Similarly, RNAi of PIAS or Smt3 decreased Smo accumulation (Fig. 2E,F). We further found that the expression of a *UAS-Ulp1* transgene inhibited Smo accumulation in wing disc (Fig. 2G), indicating that Ulp1 played a negative role in regulating Smo, which was consistent with the finding that RNAi of Ulp1 expression reduced the dominant negative activity of Smo^DN^ in the wing (Fig. 1I). The severe or mild changes in Ci accumulation and *ptc*-*lacZ* expression (Fig. 2E–G, red and green panels) may not solely reflect the changes in Smo activity because it has been shown that Ci undergoes sumoylation regulation^30^,^31^, and because Cos2 also undergoes sumoylation that regulates its activity (see below).

**Figure 3:**
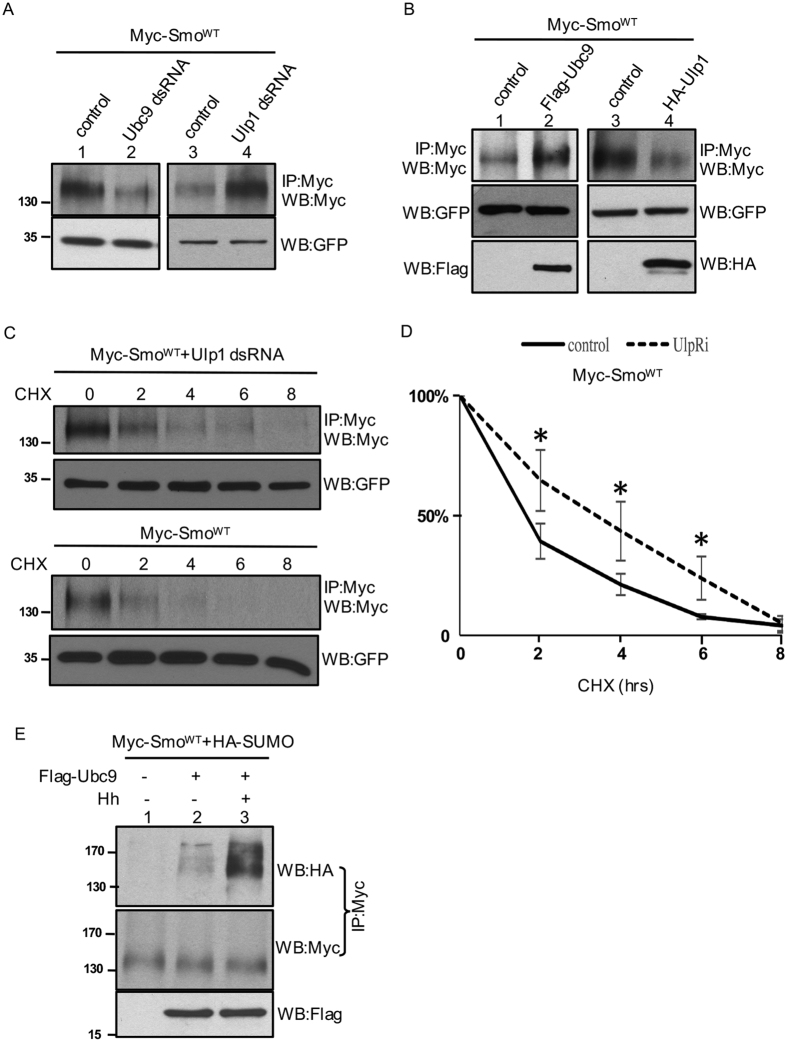
Sumoylation stabilizes Smo. (**A**) S2 cells were transfected with Myc-Smo^WT^ and treated with Ubc9 dsRNA, Ulp1 dsRNAi, or control ds RNA. Cell extracts were subjected for immunoprecipitation and western blots with the anti-Myc antibody to detect the levels of Smo expression. Western blots with the anti-GFP antibody served as transfection and loading control. (**B**) S2 cells were transfected with Myc-Smo^WT^ along or together with Flag-Ubc9 or HA-Ulp1. Cell lysates were immunoprecipitated and western blotted with the anti-Myc antibody to detect the levels of Smo. Western blots with the anti-GFP antibody served as transfection and loading control. The expression of Ubc9 and Ulp1 was monitored by western blots with the anti-Flag and the anti-HA antibody, respectively. (**C**) Smo protein stability assay. S2 cells were transfected with Myc-Smo^WT^, treated with or without Ulp1 dsRNA, and incubated with CHX for the indicated times. Cell lysates were immunoprecipitated with the anti-Myc antibody and western blotted with the anti-Myc antibody to examine the levels of Myc-Smo^WT^. GFP western blot serves as transfection and loading control. (D) The quantification of Myc-Smo^WT^ levels at different time points. Signal density at t = 0 was defined as 100%, for either Myc-Smo^WT^ or Myc-Smo^WT^ combined with Ulp1 dsRNA. Data from four independent experiments. *p < 0.05 versus control (Student’s t test). (**E**) A sumoylation assay to detect the levels of Smo sumoylation. S2 cells were cotransfected with Myc-Smo^WT^ and HA-SUMO, with or without Flag-Ubc9 in the absence or presence of Hh. Cell lysates were immunoprecipitated with the anti-Myc antibody and western blotted with the anti-HA antibody to examine the Smo-bound SUMO. Immumuprecipitated products were also western blotted with the anti-Myc antibody to monitor the levels of Smo. Cell lysates were also western blotted to examine the expression of Ubc9. Smo was normalized by the methods described (See Materials and Methods).

In addition to examining Smo accumulation in wing discs, we further carried out a protein stability assay to determine the levels of Smo regulated by sumoylation pathway proteins. We found that inactivation of Ubc9 decreased, whereas inactivation of Ulp1 increased the levels of Smo in S2 cells (Fig. 3A). In contrast, the overexpression of Ubc9 increased, whereas the overexpression of Ulp1 decreased the levels of Smo (Fig. 3B). RNAi or overexpression of the other two desumoylases in *Drosophila*, Velo and CG12717^32^, did not change the levels of Smo in S2 cells (not shown), indicating the specificity of Ulp1 in regulating Smo. To examine Smo stability regulated by Ulp1 and more precisely examine the stability of Smo protein, we carried out a time course experiment. We transfected S2 cells with Myc-Smo^WT^ and monitored Smo levels at different time points after the treatment with the protein synthesis inhibitor cycloheximide (CHX). We performed western blots to determine the stability of the immunoprecipitated Myc-Smo^WT^ and found that the half-life of Myc-Smo^WT^ was increased by the inactivation of Ulp1 (Fig. 3C, top panels), compared to the half-life of Smo^WT^ in cells without Ulp1 inactivation (Fig. 3C, lower panels). This was further demonstrated by a quantification analysis (Fig. 3D). These data support the idea that sumoylation enhances the stability of Smo and promotes the accumulation of Smo in wing disc.

To determine whether Smo indeed undergoes sumoylation, we performed a sumoylation assay by transfecting S2 cells with the epitope-tagged Smo and SUMO, followed by immunoprecipitation with one epitope tag and western blot with another tag. We found that the overexpression of Ubc9 elevated the levels of Smo sumoylation, which was further increased by Hh treatment (Fig. 3E). This finding suggests that the sumoylation of Smo is indeed regulated by SUMO pathway proteins and that Hh activates Smo by promoting Smo sumoylation.

### Hh promotes the sumoylation of Smo independent of phosphorylation

**Figure 4:**
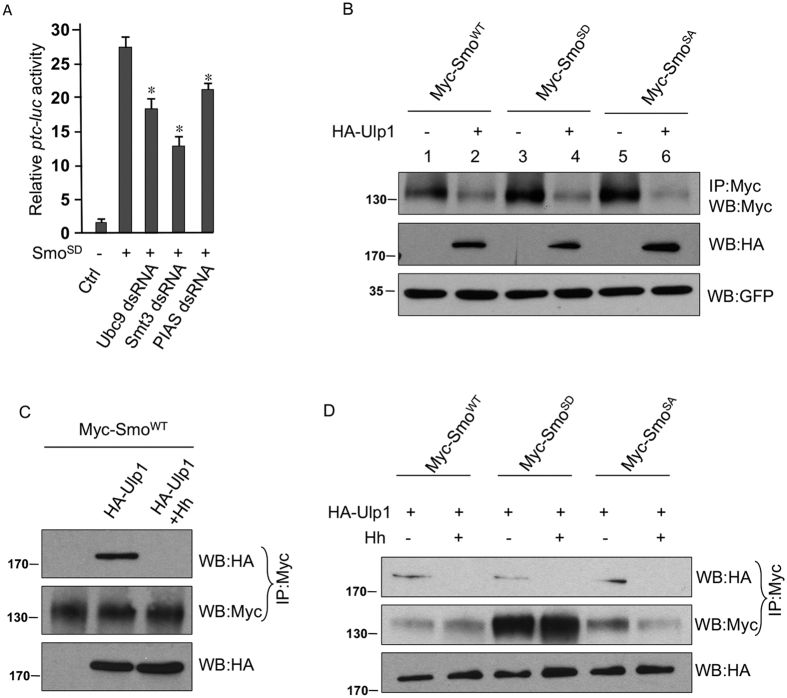
Smo sumoylation regulated by Hh does not depend on phosphorylation. (**A**) S2 cells were cotransfected with Myc-Smo^SD^ plus tub-Ci and treated with the indicated dsRNA, followed by an analysis of the *ptc-luc* reporter activity. *p < 0.001 versus high level of *ptc-luc* activity induced by the expression of Smo^SD^ in the second column (Student’s t test). (**B**) S2 cells was transfected with Myc-Smo^WT^, Myc-Smo^SD^, or Myc-Smo^SA^ in combination with or without HA-Ulp1. Cell lysates were immunoprecipitated with the anti-Myc antibody and western blotted with the anti-Myc antibody to examine Smo stability. Cell lysates were also subjected to western blot with the anti-HA antibody to monitor the expression of Ulp1. Lysates western blotted with GFP served as transfection and loading control. (**C**) S2 cells were transfected with Myc-Smo^WT^ alone or together with HA-Ulp1 in the presence or absence of Hh. Cell lysates were immunoprecipitated with the anti-Myc antibody and western blotted with the anti-HA antibody to detect Smo-bound Ulp1, or western blotted with the anti-Myc antibody to examine the levels of Smo. Smo was normalized by the methods described (See Materials and methods). Cell lysates were also western blotted with the anti-HA antibody to examine Ulp1 expression. (**D**) S2 cells were transfected with Myc-Smo constucts along with HA-Ulp1, followed by the treatement with HhN-conditioned medium or control medium. Cell lysates were immunoprecipitated with the anti-Myc antibody and western blotted with the anti-HA antibody to detect Smo-bound Ulp1, or western blotted with the anti-Myc antibody to examine the levels of Smo. Cell lysates were also subjected to western blot to examine HA-Ulp1 expression.

The activity of most phosphomimetic mutant forms of Smo can still be upregulated by Hh stimulation, raising the possibility for Smo to be regulated beyond phosphorylation^16^,^28^. To test this hypothesis, we transfected S2 cells with Myc-Smo^SD^, a phosphomimetic form of Smo in which PKA and CK1 sites in the three phosphorylation clusters were mutated to aspartate (previously we named it Smo^SD123^), and treated cells with dsRNA targeting Ubc9, Smt3, and PIAS. We found that the *ptc-luc* activity of Smo^SD^ was significantly reduced by RNAi of Ubc9, Smt3, and PIAS (Fig. 4A). Using the immunoprecipitation assay to examine the levels of Smo expression in cultured S2 cells, we further found that the stability of Smo^WT^, Smo^SD^, and Smo^SA^ (a phosphorylation deficient form of Smo bearing Ser to Ala mutations in clusters of PKA and CK1 sites) were all decreased by the overexpression of Ulp1 desumoylase (Fig. 4B). These data suggest that Smo stability is regulated by sumoylation regardless of phosphorylation.

To explore the mechanisms of Smo regulation by sumoylation, we examined the physical interaction between Smo and its desumoylase Ulp1. We found that Myc-Smo^WT^ was associated with HA-Ulp1 in the immunoprecipitation assay (Fig. 4C). In addition, the treatment of Hh severely reduced Smo-Ulp1 interaction (Fig. 4C), indicating that Hh promotes Smo sumoylation by disassociating the desumoylase. We further explored the possibility of Ulp1 interaction with other forms of Smo. We found that Ulp1 physically interacted with the wild-type, phosphomimetic, and phosphorylation-deficient forms of Smo, and such interaction was attenuated by Hh stimulation (Fig. 4D). This finding explains why the stability of all forms of Smo was decreased by Ulp1 expression.

### Krz destabilizes Smo through blocking Smo sumoylation

**Figure 5:**
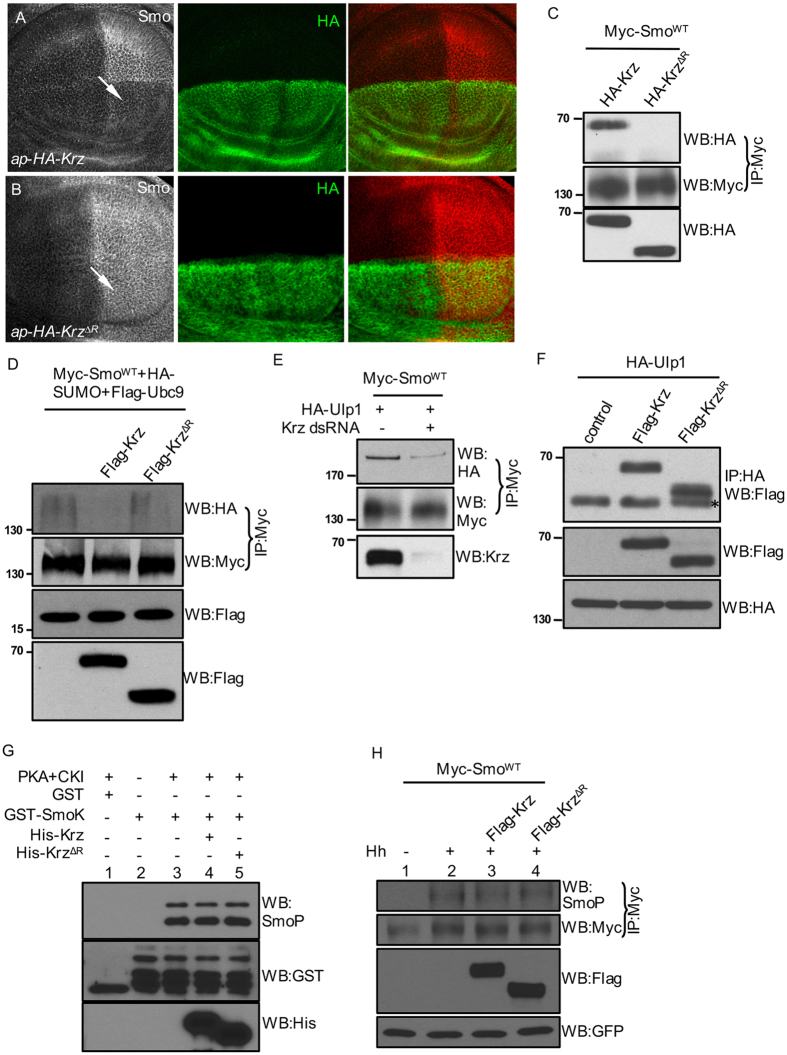
Krz inhibits Smo accumulation through preventing Smo sumoylation. (**A,B**) Wing discs expressing HA-Krz or HA-Krz^∆R^ by the dorsal compartment-specific *ap*-Gal4 were stained for Smo and HA. Arrow in A indicates Smo accumulation severely decreased by HA-Krz. Arrow in B indicates the unaffected Smo accumulation. (**C**) S2 cells were transfected with Myc-Smo^WT^ and either HA-Krz or HA-Krz^∆R^. Cell lysates were immunoprecipitated with the anti-Myc antibody, followed by western blot with the anti-HA antibody to examine the Smo-bound Krz. Cell lysates were also western blotted to examine the expression of Krz or Krz^∆R^. (**D**) Myc-Smo^WT^ and HA-SUMO were cotransfected into S2 cells with HA-Krz or HA-Krz^∆R^, followed by immunoprecipitation with the anti-Myc antibody and western blot with the anti-HA antibody to examine Smo sumoylation, or with the anti-Myc antibody to monitor the level of Smo. Cell lysates were also western blotted to examine the expression of Krz or Krz^∆R^. (**E**) S2 cells were cotransfected with Myc-Smo^WT^ and HA-Ulp1, treated with Krz dsRNA or control dsRNA. Immunoprecipitates were western blotted with the anti-HA antibody to examine the Smo-bound Ulp1, or with the anti-Myc antibody to examine the levels of Smo. Cell lysates were western blotted with the anti-Krz antibody to examine the endogenous Krz expression. (**F**) S2 cells were transfected with HA-Ulp1 and either Flag-Krz or Flag-Krz^∆R^, followed by immunoprecipitation with the anti-HA antibody and western blot with the anti-Flag antibody to examine the Ulp1-bound Krz. The expression of Krz and Krz^∆R^ was examined by western blot with cell lysates. The asterisk in top panel indicates the mouse IgG. (**G**) *in vitro* kinase assay to examine Smo phosphorylation. GST or GST-Smo was incubated with recombinant PKA and CK1 kinases with either His-Krz or His-Krz^∆R^. Western blot with the anti-SmoP antibody examines the phosphorylation of Smo. (**H**) S2 cells were transfected with the indicated constructs followed by the treatment with HhN-conditioned medium of control medium. Immunoprecipitates were western blotted with the anti-SmoP antibody to detect the levels of Smo phosphorylation, or with the anti-Myc antibody to monitor the levels of Smo. In C, D, and H, Smo normalization described in Materials and Methods.

It has been shown that the excessively expressed Krz prevents Smo accumulation, although Smo has no change in *krz* mutant cells^15^,^22^. In this study, we carried out experiments to determine how Krz blocks the accumulation and prevents the activation of Smo. Domain functions of β-arrestin have not been well characterized, although β-arrestin interacts with many protein partners and exhibits conformational changes during cell signaling^33^. The C-terminal regulatory domain consisting of 44 amino acids is highly conserved among β-arrestins^34^,^35^. We found that expression of Krz^ΔR^ lacking the regulatory domain did not prevent Smo accumulation in wing discs (Fig. 5B, compared to Fig. 5A). We also found that Krz, but not Krz^ΔR^, interacted with Smo in an immunoprecipitation assay (Fig. 5C). These data suggest that the C-terminal regulatory domain is required for Krz to inhibit Smo accumulation.

To further investigate the role of Krz in regulating Smo, we transfected S2 cells with Myc-Smo^WT^ with either Krz or Krz^ΔR^ to determine whether Krz regulates Smo sumoylation. We found that the overexpression of Flag-Krz, but not Flag-Krz^ΔR^, blocked Smo sumoylation. One possibility was that Krz facilitated Ulp1 to desumoylate Smo. To test this, we cotransfected S2 cells with Myc-SmoWT and HA-Ulp1, treated cells with or without Krz dsRNA, and used the co-immunoprecipitation assay to examine the association of Smo and Ulp1. We found that RNAi of Krz decreased the interaction between Smo and Ulp1, suggesting that Krz facilitates Smo-Ulp1 interaction (Fig. 5E). We then examined whether Krz was associated with Ulp1 in cultured S2 cells. Interestingly, both Krz and Krz^ΔR^ physically interacted with Ulp1 in the immunoprecipitation assay (Fig. 5F). Considering Krz^ΔR^ not interacting with Smo (Fig. 5C), our data suggest that Ulp1 and Smo interact with Krz through different domains, which support the idea that Krz inhibits Smo accumulation by facilitating Ulp1 to interact with and desumoylate Smo.

Given the fact that Hh activates Smo by promoting both phosphorylation and sumoylation of Smo, and the fact that Krz inhibits Smo sumoylation in cultured cells (Fig. 5D) and blocks Smo accumulation in wing disc (Fig. 5A), we wondered whether it was possible for Krz to regulate Smo phosphorylation. Using an *in vitro* kinase assay, we found that Krz and Krz^ΔR^ did not inhibit Smo phosphorylation detected by a phospho-Smo antibody (Fig. 5G). Consistently, Krz and Krz^ΔR^ did not inhibit Smo phosphorylation in cultured S2 cells (Fig. 5H). These results indicate that Krz inhibits Smo activation by specifically preventing Smo sumoylation, but not phosphorylation.

### The inhibitory role of Cos2 on Smo is attenuated by sumoylation

**Figure 6:**
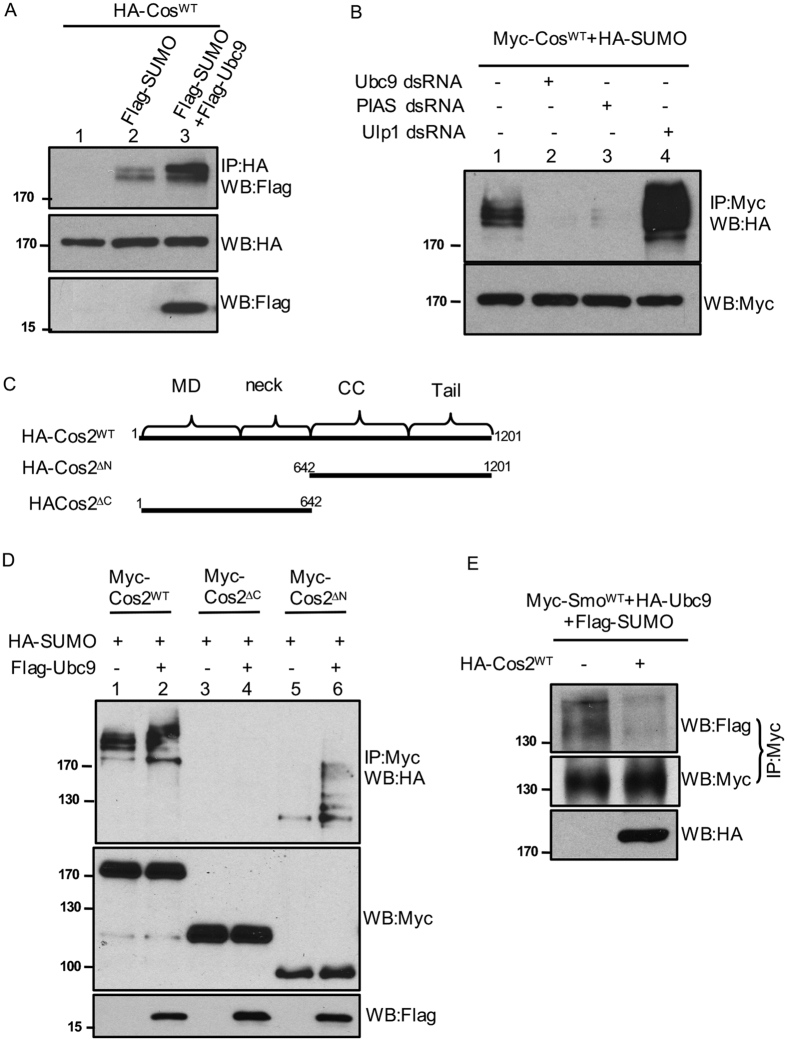
Sumoylation attenuates the inhibitory activity of Cos2 on Smo. (**A**) S2 cells were transfected with HA-Cos2^WT^ alone or together with Flag-SUMO, or Flag-Ubc9 plus Flag-SUMO. Cell lysates were immunoprecipitated with the anti-HA antibody followed by western blotting with the anti-Flag antibody to examine Cos2 sumoylation (bands above the 170 kd marker). Cell lysates were also blotted with the anti-HA antibody to monitor Cos2 expression (bands around the 170 kd marker), and with the anti-Flag antibody to examine Ubc9 expression (band above the 15 kd marker). (**B**) S2 cells were cotransfected with Myc-Cos2^WT^ and HA-SUMO, treated with control dsRNA, Ubc9 dsRNA, PIAS dsRNA, or Ulp1 dsRNA. Cell lysates were immunoprecipitated with the anti-Myc antibody and western blotted with the anti-HA antibody to examine Cos2 sumoylation. Lysates were also western blotted with the anti-Myc antibody to examine the levels of Cos2 expression. (**C**) A schematic drawing of Cos2 full-length and its truncations. (**D**) S2 cells were transfected with the indicated Myc-tagged Cos2 construct combined with HA-SUMO, with or without Flag-Ubc9. Cell lysates were immunoprecipitated with the anti-Myc antibody followed by western blot with the anti-HA antibody to examine the Cos2-bound SUMO. Cell lysates were also subjected to western blot with the anti-Myc antibody to monitor the levels of Cos2 expression. (**E**) To determine whether Cos2 regulates Smo sumoylation, S2 cells were transfected with the indicated constructs followed by immunoprecipitation with the anti-Myc antibody to pull down Smo. Western blots were carried out using the anti-Flag antibody to examine Smo-bound SUMO, and using the anti-Myc antibody to monitor Smo expression. Cell lysates western blotted with the anti-HA antibody indicate the expression of Cos2. Smo normalization was described in Materials and Methods.

When we examined Smo sumoylation in S2 cells using the immunoprecipitation assay, we also discovered that other components in Hh signaling cascade were sumoylated. As shown in Fig. 6A, the sumoylated HA-tagged Cos2 was detected by an anti-Flag antibody in S2 cells cotransfected with HA-tagged Cos2 and Flag-tagged SUMO (Fig. 6A, lane 2, top panel). Similar to the pattern of Smo sumoylation, the Flag signal exhibited lower mobility shifts compared to the major band detected by the anti-HA antibody (Fig. 6A, lane 2, middle panel), indicating that these bands correspond to sumoylated forms of Cos2. Overexpression of Ubc9 caused an increase in the levels of Cos2 sumoylation, especially the stronger signal in the band with lowest mobility shift (Fig. 6A, lane 3, top panel). To further characterize Cos2 sumoylation, we transfected S2 cells with Myc-tagged Cos2 and treated cells with dsRNA targeting Ubc9, PIAS, or Ulp1. The ladder pattern of Flag signals was severely decreased by either Ubc9 or PIAS RNAi (Fig. 6B, lane 2 and 3, top panel), however increased by Ulp1 RNAi (Fig. 6B, lane 4, top panel). These data suggest that Cos2 undergoes sumoylation that is regulated by the same sets of proteins involved in Smo sumoylation. We further narrowed down the sumoylated region in Cos2. As shown in Fig. 6C,D, the N-terminus of Cos2 containing the microtubule-binding domain and the neck domain barely exhibited any sumoylation, whereas Cos2 C-terminus containing the coiled-coil and C-tail domains was sumoylated, indicated by the ladder pattern of sumoylation in the western blot with the immunoprecipitated Cos2.

We have previously shown that Cos2 exhibits an inhibitory role on Smo phosphorylation and accumulation^36^. We therefore questioned the possibility for Cos2 to regulate Smo sumoylation. We thus transfected S2 cells with Myc-Smo^WT^ and HA-Cos2^WT^ and carried out immunoprecipitation assay to examine Smo sumoylation. We found that Cos2 blocked Smo sumoylation induced by the expression of Ubc9 (Fig. 6E), suggesting that Cos2 regulates the phosphorylation and sumoylation of Smo, adding an additional layer feedback regulation of Smo by Cos2.

**Figure 7:**
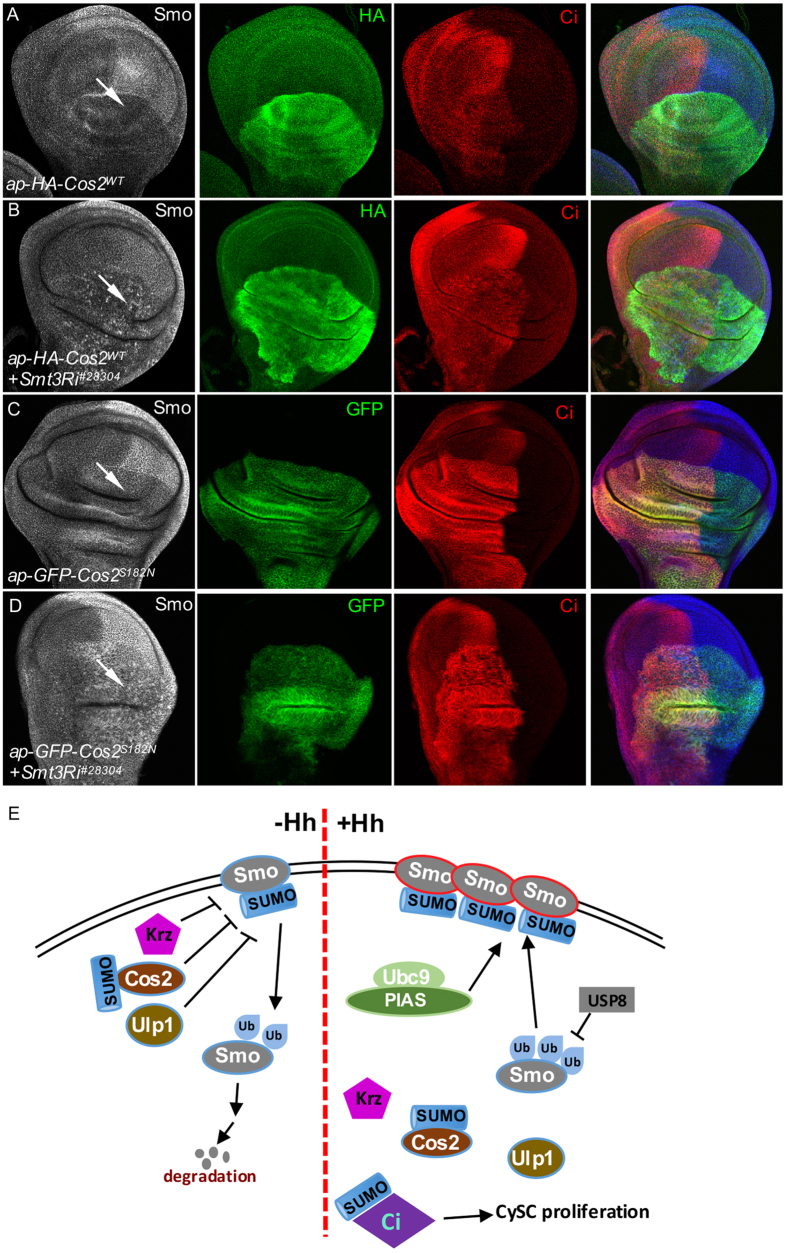
Sumoylation of Cos2 counteracts its inhibitory activity toward Smo. (**A,B**) Wing discs from third instar larvae expressing HA-Cos2^WT^ with or without Smt3RNAi by the *ap*-Gal4 were immunostained for Smo, HA, and Ci. Arrow in C indicates Smo accumulation inhibited by Cos2 expression. Arrow in D indicates the partially restored Smo accumulation by RNAi of Smt3. (**C,D**) Wing discs expressing GFP-Cos2^S182N^ with or without Smt3RNAi by the *ap*-Gal4 were immunostained for Smo, HA, and Ci. Arrow in C indicates Smo accumulation inhibited by Cos2^S182N^ expression. Arrow in D indicates the partially restored Smo accumulation by RNAi of Smt3. Of note, Smo exhibited punctate pattern when Cos2 and Smt3 RNAi were coexpressed by the *ap*-Gal4. (**E**) A model for sumoylation to activate Smo. In the absence of Hh, Smo is ubiquitinated and internalized for degradation. Krz, Cos2, and Ulp1 downregulate Smo stability through inhibiting Smo sumoylation. In the presence of Hh, Ubc9 and PIAS sumoylation proteins promote sumoylation and therefore the accumulation and activation of Smo on the membrane. In turn, sumoylation of Smo recruits USP8 deubiquitinase to inhibit Smo ubiquitination and degradation. Cos2 sumoylation attenuates its ability to inhibit Smo. Hh stimulation reduces the interaction between Smo and Ulp1. Ci is directly sumoylated by the sumoylation proteins, which promotes Ci activity in regulating CySC proliferation in the testis.

To determine whether Cos2 sumoylation regulates its inhibitory activity on Smo accumulation, we turn to the *Drosophila* imaginal disc to examine the levels of Smo accumulation. Consistent with our previous findings^36^, the expression of Cos^WT^ blocked Smo accumulation in posterior compartment cells (Fig. 7A). Interestingly, coexpression of Cos2^WT^ with Smt3 RNAi partially rescued Smo accumulation, although the accumulated Smo exhibited a punctate staining pattern (Fig. 7B). Changing the Ser182 of Cos2 to Asn (S182N) in the P-loop gave rise to a dead or a dominant-negative form^37^. Interestingly, the expression of Cos2^S182N^ also blocked Smo accumulation in the wing disc (Fig. 7C), suggesting that the inhibitory role of Cos2 on Smo is independent of Cos2 activity, raising the possibility that Cos2 inhibits Smo through direct association between Cos2 and Smo. Moreover, coexpressing Cos2^S182N^ with Smt3 RNAi also partially rescued Smo puncta accumulation (Fig. 7D). These data suggest that Cos2 sumoylation may antagonize its inhibitory role to block Smo phosphorylation and sumoylation.

## Discussion

The intracellular trafficking of Smo has been speculated as the critical step in Hh-stimulated Smo accumulation and activation. Smo cell surface accumulation and activation are promoted by phosphorylation that triggers Smo dimerization, and inhibited by ubiquitination that mediates Smo endocytosis^10^,^17^. Although a recent study showed that cell surface accumulation of Smo requires sumoylation, which antagonizes its ubiquitination-mediated endocytosis^31^, whether and how the intracellular Hh signaling components regulates Smo sumoylation remains unclear. In this study, we provide consistent evidence that SUMO pathway enzymes regulate the stability and activity of Smo. Importantly, we show that Krz, through its regulatory domain, inhibits Smo by preventing Smo sumoylation but not phosphorylation. We further show that Cos2 is also sumoylated, which antagonizes its inhibitory activity on Smo accumulation. We provide a model for Smo regulation by sumoylation and its interacting proteins including Krz and Cos2 (Fig. 7E). The mechanisms of Smo regulation by sumoylation and other intracellular signaling components may provide novel insights in the regulation of other GPCR family members.

It would be of great interest if the sumoylation residue(s) were identified in Smo. In the preparation of this manuscript, an independent study was published in which Lys851 was identified as a critical sumoylation site in Smo C-tail^31^. The same study also found that mutating Lys851 to Ala decreased Smo stability and downregulated Smo activity^31^. Although we mutated Lys851 to Ala in order to block sumoylation at this residue and carried out immunoprecipitation assay to determine the levels of Smo sumoylation in S2 cells, we still observed high levels of sumoylation in Smo (data not shown). It is possible that other residues in Smo are also sumoylated, which may play similar roles in regulating the stability and signaling activity of Smo.

To identify the sumoylation residue(s) in Cos2, we carried out immunoprecipitation assays using a series of Cos2 truncations as previously described^36^. We found that the microtubule binding domain and the neck domain of Cos2 did not undergo any sumoylation. In contrast, the coiled-coil domain and C-tail were sumoylated (Fig. 6D). However, mutating four lysine residues (Lys715, Lys892, Lys922, Lys979) in the coiled-coil domain did not affect Cos2 sumoylation. It has been shown that Cos2 C-tail is responsible for Cos2 to inhibit Smo^36^, however Cos2 still underwent the same levels of sumoylation when Lys1083 in the C-tail was mutated. Furthermore, mutating these lysine residues did not change the ability of Cos2 to inhibit Smo sumoylation. We speculate that Cos2 is sumoylated at other lysine residues.

In this study, we found that both the wild-type Cos2^WT^ and dead form Cos2^S182N^ inhibited Smo accumulation in wing disc (Fig. 7A,C), suggesting that Cos2 inhibits Smo accumulation in a Cos2-activity-independent manner. In addition, the inactivation of Smt3, the single form of SUMO in *Drosophila*, counteracted the inhibitory activity of Cos2^WT^ and Cos2^S182N^ in regulating Smo accumulation in the wing (Fig. 7B,D), indicating the inhibitory activity of Cos2 is regulated by the SUMO pathway. Interestingly we found that Smo was partially recovered as many tiny puncta in the wing (Fig. 7B,D). This punctate pattern suggests that Smo is located in the intracellular compartments, and these forms of Smo are likely in their inactive states. It is possible that these forms of Smo are not sumoylated because Smt3 is inactivated by RNAi. The downregulation of Smo sumoylation by RNAi of Smt3 likely prevent Smo accumulation and activation in the wing disc, although the inhibitory activity of Cos2 is compromised. Future studies could be designed to examine the co-localization of Smo with intracellular compartments labeled by different markers, which will provide a better understanding Smo intracellular trafficking. Multiple Hh signaling components are sumoylated, resulting in difficulty analyzing the phenotypes in adult wings and wing discs. For example, Ci is sumoylated, which promotes the activity of Ci in regulating cyst stem cell (CySC) proliferation^30^ (Fig. 7E). However, we are able to distinguish the roles of Smo and Cos2 sumoylation using both the wing disc phenotypes and cell cultured assays to examine the levels of protein sumoylation.

The other very critical finding in this study is that Krz prevents Smo accumulation by inhibiting the sumoylation of Smo. More importantly, the regulatory domain of Krz is responsible to inhibit Smo sumoylation, however, none of the Krz forms regulate Smo phosphorylation. These data suggest the specificity of Krz in regulating Smo sumoylation, accumulation, and ultimate activation. Our observation is in line with the previous finding in which Krz mediated Smo degradation partially through the proteasome pathway in an ubiquitination independent manner^15^. However, our data presented in this study and those from the previous study^15^ did not exclude the possibility for Krz to downregulate Smo accumulation and activation through another intracellular signaling pathway. In this study, we found that the excessively expressed Krz prevents Smo sumoylation likely by facilitating Ulp1 to interact with Smo. However, it should be noted that the loss of endogenous Krz does not cause changes in Smo accumulation in wing disc. It might be possible that other arrestins, such as arrestin-1 and arrestin-2 in *Drosophila*, compensate the loss of function of Krz. We found that RNAi of Krz decreased the interaction between Smo and Ulp1 (Fig. 5E). Although the RNAi of Krz was very efficient, there was still weak interaction between Smo and Ulp1 (Fig. 5E), raising the possibility that other arrestins might have similar roles.

## Materials and Methods

### Constructs, mutants, transgenes

Myc-Smo^WT^, Myc-Smo^SD^ (i.e. Smo^SD123^), Myc-Smo^SA^ (i.e. Smo^-PKA123^), Flag-Smo^DN^ (i.e. Smo^−PKA12^), HA-Cos2^WT^, HA-Cos2^∆C^, and HA-Cos2^∆N^ constructs and transgenic lines have been previously described^28^,^36^. HA-tagged Ulp1 (HA-Ulp1), HA-tagged Velo (HA-Velo), and HA-tagged CG12717 (HA-CG12717 were gifts from Dr. Liqun Luo^32^. Epitope-tagged Krz and Krz^∆R^ were PCR amplified from cDNA clone (LD31082) and sub-cloned into UAST-2x HA or UAST-2xFlag vectors within *NotI* and *KpnI* sites. HA-SUMO and Flag-SUMO were generated by inserting SUMO cDNA (PCR amplified from clone LD07775) to the UAST-5xHA and UAST-2xFlag vector, respectively. Flag-PIAS were constructed by inserting PIAS cDNA (clone LD27861) to UAST-2xFlag vector. Myc-Cos2^WT^, Myc-Cos2^∆C^, and Myc-Cos2^∆N^ were generated by insertion of Cos2 full-length or Cos2 fragments (from HA-Cos2^∆C^, and HA-Cos2^∆N^) into the UAST-5xMyc vector. His-Krz and His-Krz^ΔR^ contained Krz amino acids 1-470 or 1-426 lacking the regulatory domain. Transgenic Ubc9 (CG3018) RNAi lines were obtained from either BSC (#31396) or VDRC (v33685), and line v33685 was used for most of the experiments as these lines gave rise to similar phenotypes. PIAS [CG8068, Su(var)2-10] RNAi lines (#31623 and #29448) and Smt3 (CG4494) RNAi line (#28034) were obtained from BSC. Ulp1 (CG12359) RNAi lines were from BSC (#31624), VDRC (v31744), and Dr. Alexey Veraksa^38^. The line v31744, combined with Dicer co-expression, was used for most of the experiments. Velo (CG10107) RNAi line (v103524) was from VDRC. Flag-PIAS, Flag-Ubc9, HA-Krz, and HA-Krz^ΔR^ transgenic lines were generated using the 75B1 attP locus^39^. HA-Ulp1 and GFP-Cos2^S182N^ transgenic lines were obtained from BSC. *MS1096*-Gal4, *ap-*Gal4, and *C765*-Gal4 have been described^40^,^41^.

### Cell culture, transfection, immunoprecipitation, and western blot

S2 cell culture and transfection using Effectene transfection reagent (Qiagen) has been previously described^16^. Forty-eight hours post-transfection, cells were harvested and treated with lysis buffer [100 mM NaCl, 50 mM Tris-HCl (pH8.0), 1.5 mM EDTA, 10% (vol/vol) glycerol, 1% (vol/vol) Nonidet P-40, and protease inhibitor tablet (Roche)]. Cell lysate was obtained by centrifugation at 12,000 rpm for 10 min. A total of 6 × 10^6^ cells were harvested and lysed in 450 μL lysate buffer. 50 μL was saved for direct western blots, out of which 4 μL was used for each load. The remaining 400 μL was used for IP assay. The cell lysate was added with beads of Protein A Ultralink Resin (Thermo Scientific) after adding the proper primary antibody for 2 h. The samples were then resolved by SDS-PAGE and transferred onto PVDF membranes (Millipore) for western blot. About 16 times more of the immunoprecipitation sample was analyzed compared with the corresponding lysate. Western blot analysis was performed using the indicated antibodies and the enhanced chemiluminescence (ECL) protocol. To normalize the levels of Smo, 50 μM MG132 (a proteasome inhibitor, Calbiochem) and 15 mM NH_4_Cl (a lysosome inhibitor, Sigma-Aldrich) were used to block Smo degradation, and samples were normalized for loading^14^,^16^. For Smo stability assay, Cycloheximide (Sigma) treatment was performed at a final concentration of 100 μM for the indicated time points before harvesting S2 cells^36^. Density of the western blot was analyzed by ImageJ software. Hh treatment achieved by transfection with HhN cDNA combined with treatment with 60% of HhN-conditioned medium to achieve high level of Hh signaling activity, and RNAi achieved by adding dsRNA to cell culture in 6-well plates have been previously described^16^,^39^. GFP dsRNA was used as previous described^14^. Ulp1 dsRNA was synthesized against coding sequence 171–720, Velo dsRNA was against 2100–2640, CG12717 dsRNA was against 1–500, PIAS dsRNA was against 131–680, Krz dsRNA against 192–798, Smt3 and Ubc9 dsRNAs were against full coding sequence plus 3′-UTR. Antibodies used for western blotting: mouse anti-Myc (9E10, Santa Cruz, 1:5,000), anti-HA (F7, Santa Cruz, 1:5,000), anti-Flag (M2, Sigma, 1:10,000), and anti-GFP (Millipore, 1:1,000); rabbit anti-Krz (Thermo Scientific, 1:2,000). The consistency of western blots was confirmed by three to five individual repeats.

### *In vitro* kinase assay, RT-PCR, and luciferase reporter assay

For *in vitro* kinase assay, GST-SmoK containing aa656–753 of Smo, His-Krz, and His-Krz^∆R^ fusion proteins were expressed in bacteria and purified with GST beads. 3 μg of Smo were incubated at 30 °C for 30 min in 50 μL of assay buffer (20 mM Tris-HCl at pH8.0, 10 mM MgCl_2_, 0.2 mM EDTA, 1 mM DTT), and 2.5 μM ATP in the presence of commercial recombinant PKA and CK1 (New England Biolabs) followed by western blotting with antibodies to examine Smo phosphorylation. Antibody used: rabbit anti-GST (Santa Cruz, 1:500), anti-SmoP (1:20)^16^, mouse anti-His (H8, Millipore, 1:1,000).

To examine the levels of gene expression, RT-PCR was carried out using S2 cells with the primers for Ubc9 (5′-TGG CGC AAG GAT CAC-3′; 5′-GCC CGC CCT CCC AGG-3′), PIAS (5′-CAG CTG CCT AAT GTC ATT C-3′; 5′-GAC ACC ACT GAA CCG-3′), Smt3 (5′-AGA AGG GAG GTG AGA C-3′; 5′-CGT TCA TCA GCT TCC TC-3′), and Ulp1 (5′-CGG GAT TCC AGG CTC-3′; 5′-GTC CAC ACG CCG GTA C-3′).

The *ptc-luc* reporter assay has been described with S2 cells cultured in 6-well plates and transfected with 50 ng *tub*-Ci and 150 ng *ptc-luc* reporter constructs followed by activity analysis using the Dual-Luciferase Reporter Assay System (Promega, Madison, WI, USA) combined with the GLOMAX Multi Detection System (Promega)^16^. Each *ptc-luc* experiment was repeated three times and the error bars indicated standard deviation (S.D.) from four repeats.

### Immunostaining of wing imaginal discs

Wing discs from third instar larvae with specific genotypes were dissected in PBS then fixed with 4% (vol/vol) formaldehyde in PBS for 20 min. After permeabilization with PBST [PBS supplemented with 1% (vol/vol) Triton X-100], discs were incubated with the indicated primary antibodies for 3 h and the corresponding second antibodies for 1 h, and then washed with PBT for three times, for 20 min per wash. Primary antibodies used in this study: mouse anti-SmoN (DSHB, 1:10); rabbit anti-β-Gal (Cappel, 1:1,500), anti-HA (Santa Cruz, Y-11, 1:100); rat anti-Ci (Developmental Studies Hybridoma Bank, 1:10). Secondary antibodies were from Jackson ImmunoResearch Laboratories Inc., affinity-purified for multiple labeling (1:500). Samples were mounted on slides in 80% glycerol and Fluorescence signals were acquired with the 20 x objective on an Olympus confocal microscope. The images shown represent five or more images collected for each experiment.

## Additional Information

**How to cite this article:** Zhang, J. *et al*. SUMO regulates the activity of Smoothened and Costal-2 in *Drosophila* Hedgehog signaling. *Sci. Rep.* **7**, 42749; doi: 10.1038/srep42749 (2017).

**Publisher's note:** Springer Nature remains neutral with regard to jurisdictional claims in published maps and institutional affiliations.
